# Supplementary material for: Frequency of Polymorphisms in SLC47A1 (rs2252281 and rs2289669) and SLC47A2 (rs34834489 and rs12943590) and the Influence of SLC22A1 (rs72552763 and rs622342) on HbA1c Levels in Mexican-Mestizo Patients with DMT2 Treated with Metformin Monotherapy
Source: Int J Mol Sci. 2025 Sep 5;26(17):8652. doi: 10.3390/ijms26178652 (PMC12429666; doi:10.3390/ijms26178652)
Supplement: Supplementary file 1 [file ijms-26-08652-s001.zip › Figure S1.pdf]

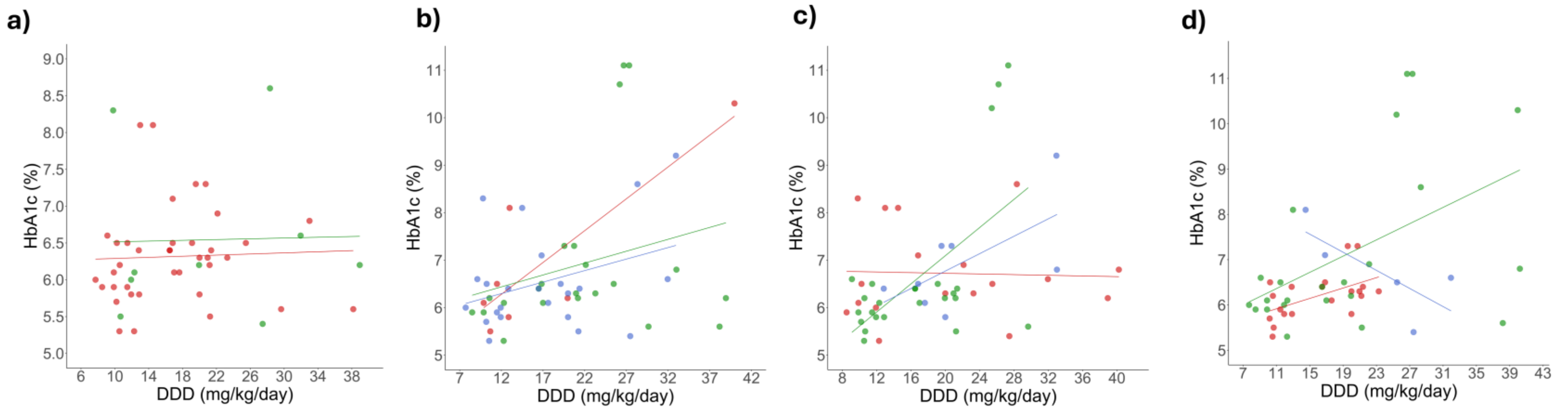

Figure S1. Effect of metformin daily dose on HbA1c levels sorted by genotypes of *SLC47A1* and *SLC47A2*. Correlation analysis between HbA1c levels and metformin dose for each genotype of: a) rs2252281 [TT (n = 38, red dots) HbA1c (%) = 6.3 + (0.003 x DDD), r^2 =0.002, p value= 0.808; TC (n = 9, green dots) HbA1c (%) = 6.5 + (0.002 x DDD), r^2 = 0.001, p value= 0.950]. b) rs2289669 [GG (n = 7, red dots) HbA1c (%) = 4.7 + (0.134 x DDD), r^2 = 0.711, p value=0.017; AG (n = 22, green dots) HbA1c (%) = 5.8 + (0.050 x DDD), r^2 = 0.062, p value= 0.261; AA (n = 23, blue dots) HbA1c (%) = 5.7 + (0.048 x DDD), r^2 = 0.118, p value= 0.108]. c) rs1294359 [GG (n = 18, red dots) HbA1c (%) = 6.8 - (0.003 x DDD), r^2 = 0.001, p value=0.887; AG (n = 25, green dots) HbA1c (%) = 4.3 + (0.141 x DDD), r^2 = 0.335, p value= 0.002; AA (n = 23, blue dots) HbA1c (%) = 4.9 + (0.091 x DDD), r^2 = 0.408, p value= 0.088]. And d) rs34834489 [GG (n = 20, red dots) HbA1c (%) = 5.3 + (0.057 x DDD), r^2 = 0.441, p value =0.016; AG (n = 23, green dots) HbA1c (%) = 5.4 + (0.089 x DDD), r^2 = 0.247, p value= 0.016; AA (n = 5, blue dots) HbA1c (%) = 9.1 - (0.100 x DDD), r^2 = 0.561, p value= 0.145].
