# Supplementary material for: Frequency of Polymorphisms in SLC47A1 (rs2252281 and rs2289669) and SLC47A2 (rs34834489 and rs12943590) and the Influence of SLC22A1 (rs72552763 and rs622342) on HbA1c Levels in Mexican-Mestizo Patients with DMT2 Treated with Metformin Monotherapy
Source: Int J Mol Sci. 2025 Sep 5;26(17):8652. doi: 10.3390/ijms26178652 (PMC12429666; doi:10.3390/ijms26178652)
Supplement: Supplementary file 1 [file ijms-26-08652-s001.zip › Table S1.pdf]

Table S1. Patients' clinical and demographic characteristics sorted by genotypes of rs2252281 and rs2289669 in *SLC47A1*.

Characteristic	rs2252281 genotype		p value	rs2289669 genotype			p value
	TT (49)	TC (10)		GG (8)	AG (24)	AA (27)	
Sex:							
Male	15 (0.306)	2 (0.200)	0.770 Chi	2 (0.250)	9 (0.375)	6 (0.222)	0.484 Chi
Female	34 (0.69)	8 (0.800)		6 (0.750)	15 (0.625)	21 (0.778)	
Age, years	55.2 ± 11.10	58.8 ± 9.30	0.295 t	57.8 ± 8.24	53.8 ± 11.2	57.0 ± 11.2	0.553 1
Dichotomic age:							
<55	23 (0.469)	3 (0.300)	0.526 Chi	2 (0.250)	12 (0.500)	12 (0.444)	0.467 Chi
≥55	26 (0.531)	7 (0.700)		6 (0.750)	12 (0.500)	15 (0.556)	
Height, m	1.57 ± 0.072	1.57 ± 0.058	0.851 t	1.57 ± 0.072	1.59 ± 0.076	1.55 ± 0.058	0.079 1
Weight, kg	80.2 (66.7–86.0)	75.4 (66.4–86.3)	0.694 U	76.8 (65.8–85.4)	80.2 (68.4–88.5)	79.5 (64.8–85.8)	0.450 2
BMI, kg/m^2	31.2 (27.1–35.3)	30.0 (26.7–34.4)	0.592 U	28.4 (27.6–35.3)	30.5 (28.5–33.9)	31.4 (26.6–35.8)	0.933 2
BMI classification:							
Normal weight	3 (0.061)	1 (0.100)		0 (0.000)	2 (0.083)	2 (0.74)	
Overweight	16 (0.327)	4 (0.400)		5 (0.625)	7 (0.292)	8 (0.296)	
Obese I	16 (0.327)	2 (0.200)	0.676 Chi	0 (0.000)	9 (0.375)	9 (0.333)	0.558 Chi
Obese II	9 (0.184)	3 (0.300)		2 (0.250)	5 (0.208)	5 (0.185)	
Obese III	5 (0.102)	0 (0.000)		1 (0.125)	1 (0.042)	3 (0.111)	
Systolic BP, mmHg	122.0 (111.0–136.0)	120.0 (111.0–122.0)	0.533 U	122.0 ± 17.6	125.0 ± 20.2	126.0 ± 15.2	0.873 1
Diastolic BP, mmHg	76.1 ± 11.4	72.9 ± 10.4	0.405 t	73.8 ± 12.7	75.9 ± 10.0	75.7 ±12.1	0.894 1
Treatment time, years	3.0 (1.0–8.0)	5.0 (3.0–6.8)	0.161 U	2.0 (1.4 – 4.8)	3.5 (1.0–7.3)	4.0 (2.0–8.5)	0.651 2
Dose, mg:							
≤850	18 (0.391)	4 (0.400)		5 (0.714)	6 (0.273)	11 (0.407)	
1700	20 (0.435)	3 (0.300)	0.598 Xi	1 (0.143)	10 (0.455)	12 (0.444)	0.273 Chi
2550	8 (0.174)	3 (0.300)		1 (0.143)	6 (0.273)	4 (0.148)	
NA	3	0		1	2	0	
DDD, mg/kg/día	18.4 (12.0–23.0)	23.1 (12.0–28.1)	0.487 U	12.9 (11.1–16.5)	21.1 (16.6–26.6)	17.6 (11.7–23.4)	0.239 2
[Metformin], ng/ml	241.69 (66.68–420.47)	116.76 (87.93–442.53)	0.696 U	132.16 (100.04–265.06)	248.02 (78.26–358.457)	187.18 (64.89–507.39)	0.964 2
Glycaemic control:							
Yes (HbA1c < 7.0%)	35 (0.714)	7 (0.700)	1.000 F	5 (0.625)	18 (0.750)	19 (0.784)	0.731 Chi
No (HbA1c ≥ 7.0%)	14 (0.286)	3 (0.300)		3 (0.375)	6 (0.250)	8 (0.216)	
HbA1c means, %	6.4 (5.9–7.3)	6.2 (6.0–7.9)	0.708 U	7.25 ± 1.82	6.96 ± 1.73	7.18 ± 2.16	0.899 1
Glucose, mg/dl	113 (102–158)	124 (112–133)	0.497 U	118 (101–195)	115 (102–138)	114 (100–161)	0.986 2

Characteristics with normal distribution are expressed through the mean plus the standard deviation; characteristics without normal distribution are expressed as the median accompanied by the interquartile range; nominal characteristics are reported as frequency (proportion). BMI: body mass index, BP: blood pressure, HbA1c: glycated hemoglobin, DDD: defined daily dose. The superscript in the p value indicates that it comes from a 1: ANOVA test, 2: Kruskal-Wallis's test, Chi: Chi-square test, U: Mann-Whitney test, t: Student's t-test, or F: Fisher test.
