# Supplementary material for: Frequency of Polymorphisms in SLC47A1 (rs2252281 and rs2289669) and SLC47A2 (rs34834489 and rs12943590) and the Influence of SLC22A1 (rs72552763 and rs622342) on HbA1c Levels in Mexican-Mestizo Patients with DMT2 Treated with Metformin Monotherapy
Source: Int J Mol Sci. 2025 Sep 5;26(17):8652. doi: 10.3390/ijms26178652 (PMC12429666; doi:10.3390/ijms26178652)
Supplement: Supplementary file 1 [file ijms-26-08652-s001.zip › Table S2.pdf]

Table S2. Patients' clinical and demographic characteristics sorted by genotypes of the rs12943590 and rs34834489 in *SLC47A2*.

Characteristic	rs12943590 genotype			p value	rs34834489 genotype			p value
	GG (20)	AG (30)	AA (9)		GG (28)	AG (26)	AA (5)	
Sex:								
Male	4 (0.200)	9 (0.300)	4 (0.444)	0.406 Chi	11 (0.393)	5 (0.192)	1 (0.200)	0.240 Chi
Female	16 (0.800)	21 (0.700)	5 (0.556)		17 (0.607)	21 (0.808)	4 (0.800)	
Age, years	54.0 ± 12.3	57.7 ± 10.8	53.4 ± 6.6	0.809[1]	56.1 ± 11.8	54.8 ± 10.7	59.4 ± 5.27	0.675[1]
Dichotomic age:								
<55	10 (0.500)	10 (0.333)	6 (0.667)	0.169 Chi	12 (0.429)	12 (0.462)	2 (0.400)	0.953 Chi
≥55	10 (0.500)	20 (0.667)	3 (0.333)		16 (0.571)	14 (0.538)	3 (0.600)	
Height, m	1.56 ± 0.063	1.57 ± 0.070	1.60 ± 0.077	0.231[1]	1.6 (1.56–1.63)	1.54 (1.51–1.58)	1.54 (1.49–1.58)	**0.021[2]**
Weight, kg	77.2 ± 15.7	78.4 ± 13.6	84.0 ± 27.2	0.597[1]	81.6 (76.6–86.8)	73.0 (65.8–86.0)	61.9 (58.6–66.7)	0.081[2]
BMI, kg/m^2	31.1 (26.8–35.7)	30.8 (28.2–35.3)	30.4 (25.8–32.8)	0.822[2]	31.5 (29.2–34.7)	30.6 (26.3–35.4)	26.7 (26.1–27.9)	0.277[2]
BMI classification:								
Normal weight	1 (0.050)	2 (0.067)	1 (0.111)	0.984 Chi	1 (0.036)	2 (0.077)	1 (0.200)	0.253 Chi
Overweight	8 (0.400)	9 (0.300)	3 (0.333)		7 (0.250)	10 (0.385)	3 (0.600)	
Obese I	5 (0.250)	10 (0.333)	3 (0.333)		13 (0.464)	5 (0.192)	0 (0.000)	
Obese II	5 (0.250)	6 (0.200)	1 (0.111)		4 (0.143)	7 (0.269)	1 (0.200)	
Obese III	1 (0.050)	3 (0.100)	1 (0.111)		3 (0.107)	2 (0.077)	0 (0.000)	
Systolic BP, mmHg	125.0 ± 12.8	125.0 ± 20.3	123.0 ± 17.4	0.950[1]	130.0 ± 19.5	121.0 ± 15.3	117.0 ± 8.5	0.125[1]
Diastolic BP, mmHg	75.0 (70.0–80.8)	80.0 (70.0–80.0)	78.0 (70.0–83.0)	0.997[2]	77.1 ± 10.4	74.5 ± 12.1	71.5 ± 10.8	0.551[1]
Treatment time, years	3.0 (1.5–7.3)	4.0 (1.3–7.8)	2.0 (1.0–6.0)	0.890[2]	2.0 (1.0–6.3)	4.0 (2.0–7.0)	8.0 (3.0–14.0)	0.124[2]
Dose, mg:								
≤850	8 (0.400)	13 (0.418)	1 (0.111)	0.374 Chi	10 (0.357)	11 (0.478)	1 (0.200)	0.334 Chi
1700	8 (0.400)	9 (0.333)	6 (0.667)		12 (0.429)	7 (0.304)	4 (0.800)	
2550	4 (0.200)	5 (0.285)	2 (0.222)		6 (0.214)	5 (0.271)	0 (0.000)	
NA	0	3	0		0	3	0	
DDD, mg/kg/día	19.7 (12.2–26.9)	16.5 (11.1–21.3)	20.0 (17.6–27.8)	0.272[2]	19.5 (11.8–21.9)	17.0 (11.7–26.0)	25.5 (16.8–27.5)	0.484[2]
[Metformin], ng/ml	133.47 (87.39–302.56)	132.16 (68.87–386.28)	485.50 (236.46–725.45)	0.236[2]	102.66 (57.60–473.70)	241.69 (80.60–365.06)	316.40 (287.14–412.89)	0.480[2]
Glycaemic control:								
Yes (HbA1c < 7.0%)	13 (0.650)	24 (0.800)	5 (0.556)	0.275 Chi	21 (0.750)	18 (0.692)	3 (0.600)	0.759 Chi
No (HbA1c ≥ 7.0%)	7 (0.350)	6 (0.200)	4 (0.444)		7 (0.250)	8 (0.308)	2 (0.400)	
HbA1c means, %	6.55 (6.2–8.1)	6.2 (5.8–6.5)	6.8 (6.4–7.3)	0.104[2]	6.3 (5.9–6.9)	6.0 (5.3–6.5)	6.6 (6.5–7.1)	0.811[2]
Glucose, mg/dl	119 (102–174)	106 (102–132)	130 (99.8–178)	0.553[2]	111 (101–149)	119 (103–193)	115 (100–127)	0.846[2]

Characteristics with normal distribution are expressed through the mean plus the standard deviation; characteristics without normal distribution are expressed as the median accompanied by the interquartile range; nominal characteristics are reported as frequency (proportion). BMI: body mass index, BP: blood pressure, HbA1c: glycated hemoglobin, DDD: defined daily dose. The superscript in the p value indicates that it comes from a 1: ANOVA test, 2: Kruskal-Wallis's test, Chi: Chi-square test, or t: Student's t-test.
