# Supplementary material for: Frequency of Polymorphisms in SLC47A1 (rs2252281 and rs2289669) and SLC47A2 (rs34834489 and rs12943590) and the Influence of SLC22A1 (rs72552763 and rs622342) on HbA1c Levels in Mexican-Mestizo Patients with DMT2 Treated with Metformin Monotherapy
Source: Int J Mol Sci. 2025 Sep 5;26(17):8652. doi: 10.3390/ijms26178652 (PMC12429666; doi:10.3390/ijms26178652)
Supplement: Supplementary file 1 [file ijms-26-08652-s001.zip › Table S5.pdf]

Table S5. Multiple comparison correction using the Bonferroni method for patient characteristics across the different genotypes of the rs34834489 polymorphism.

Characteristic	Genotype	Genotype	
		GG	AG
Age	AG	1.00	-
	AA	1.00	1.00
Height	AG	0.03	-
	AA	0.23	1.00
Weight	AG	0.39	-
	AA	0.21	0.51
BMI	AG	1.00	-
	AA	0.31	1.00
Systolic BP	AG	0.21	-
	AA	0.52	1.00
Diastolic BP	AG	1.00	-
	AA	1.00	1.00
Treatment time	AG	0.61	-
	AA	0.23	0.64
DDD,	AG	1.00	-
	AA	1.00	1.00
[Metformin]	AG	1.00	-
	AA	1.00	1.00
HbA1c	AG	1.00	-
	AA	1.00	1.00
Glucose	AG	1.00	-
	AA	1.00	1.00
